# Rabies Realities: Navigating Barriers to Rabies Control in Rural Zambia—A Case Study of Manyinga and Mwansabombwe Districts

**DOI:** 10.3390/tropicalmed9070161

**Published:** 2024-07-18

**Authors:** Muma Chipo Misapa, Eugene C. Bwalya, Ladslav Moonga, Josiah Zimba, Emmanuel S. Kabwali, Mwenya Silombe, Edgar Chilanzi Mulwanda, Christopher Mulenga, Martin C. Simuunza, Hirofumi Sawa, Bernard Hang’ombe, Walter Muleya

**Affiliations:** 1 Department of Biomedical Sciences, School of Veterinary Medicine, University of Zambia, Lusaka P.O. Box 32379, Zambia; mumamisapa1@gmail.com; 2 African Centre of Excellence for Infectious Diseases of Humans and Animals, School of Veterinary Medicine, University of Zambia, Lusaka P.O. Box 32379, Zambia; martin.simuunza@unza.zm (M.C.S.); bhangombe@unza.zm (B.H.); 3 Veterinary Department, Ministry of Fisheries and Livestock, Lusaka P.O. Box 50773, Zambia; bokojay@gmail.com; 4 Department of Clinical Studies, School of Veterinary Medicine, University of Zambia, Lusaka P.O. Box 32379, Zambia; eugene.bwalya@unza.zm; 5 Department of Paraclinical Studies, School of Veterinary Medicine, University of Zambia, Lusaka P.O. Box 32379, Zambia; ladslav.moonga@unza.zm (L.M.);; 6 Department of Agriculture and Environmental Sciences, Information and Communication University, Lusaka P.O. Box 30226, Zambia; silombe@gmail.com; 7 Department of Public Health, Mwansabombwe Municipal Council, Ministry of Local Government, Mwansabombwe P.O. Box 80424, Zambia; edmuch2000@yahoo.co.uk; 8 Cactus Foundation, Lusaka P.O. Box 10101, Zambia; chrismule37@gmail.com; 9 Department of Disease Control, School of Veterinary Medicine, University of Zambia, Lusaka P.O. Box 32379, Zambia; h-sawa@ivred.hokudai.ac.jp; 10 One Health Research Center, Hokkaido University, Sapporo 001-0020, Japan; 11 International Institute for Zoonosis Control, Hokkaido University, Sapporo 001-0020, Japan; 12 Institute for Vaccine Research and Development, Hokkaido University, Sapporo 001-0021, Japan

**Keywords:** rabies, prevention, control, vaccination, barriers, determinants

## Abstract

Rabies persists as a longstanding issue in Zambia, despite being preventable. The current control measures, including dog vaccination, population control, and movement restriction, guided by ‘The Control of Dogs Act Chapter 247 of the Laws of Zambia’, have not yielded the desired impact in many areas of the country including Manyinga and Mwansabombwe districts. These two districts continue to report low dog vaccination rates, unrestricted dog movements, and escalating cases of animal and human rabies, along with dog bites. Aligned with global aspirations to achieve zero human rabies cases by 2030, this study scrutinizes the determinants and obstacles hampering the execution of rabies control initiatives in Manyinga and Mwansabombwe. Spanning approximately 11 months, this cross-sectional study gathered pre- and post-vaccination data from 301 households in Manyinga and 100 households in Mwansabombwe. Questionnaires probed knowledge, attitudes, and practices related to rabies prevention and control. A transect survey, key informant interviews, and assessment of rabies vaccination and dog bite records complemented the data collection. Findings revealed that 88.0% of respondents from both districts possessed knowledge about rabies, confirming affected species and transmission. Moreover, 76.8% in Manyinga and 88.6% in Mwansabombwe were acquainted with rabies prevention and control methods. Concerning dog owners, 89.0% were aware of rabies, 66.0% understood its prevention and control, and the majority identified bites as the primary mode of transmission. Despite the high level of knowledge recorded during the survey, the implementation of preventive measures was low, which was attributed to low levels of law enforcement by the local government authority, inadequate staffing in the veterinary department, unwillingness to pay for dog vaccinations, and unavailability of rabies vaccine at the veterinary office in both districts. Vaccination coverage stood at 64.0% in Manyinga and 21.0% in Mwansabombwe. Notably, education and occupation exhibited a positive significant association with rabies knowledge. In terms of dog bite cases, Manyinga recorded 538 dog bite cases from 2017 to June 2022, while Mwansabombwe recorded 81 dog bite and 23 jackal bite cases from 2021 to June 2022. The study underscores critical knowledge gaps in rural areas and emphasizes the imperative for enhanced public education and awareness programs, improved rabies surveillance, free mass vaccination campaigns, and community engagement to augment vaccination coverage and knowledge about rabies.

## 1. Introduction

Rabies, a zoonotic viral malady, precipitates fatal encephalitis in humans and other mammals [1,2] Annually, it claims approximately 59,000 human lives globally, with a prevalence of 56% in Asia and 44% in Africa [3,4]. The preponderance of these fatalities transpires in rural locales [5,6]. Canines persist as the principal reservoir in developing nations, while wildlife assumes this role in developed regions [7]. In the context of developing nations, optimal eradication of human rabies hinges on the strategic management of canine rabies [8].

Against this backdrop, the World Health Organization (WHO) proffers recommendations for integral components such as mass canine immunization campaigns, population control measures, restricted breeding practices, and curtailed mobility of stray canines. These interventions serve as pivotal elements in the elimination of rabies from endemic areas and the prophylaxis of dog-mediated human rabies cases [9]. Rabies has been a persistent challenge in Zambia since the 20th century [10]. It is an endemic notifiable disease in both humans and animals, with a relative prevalence hypothesized to be 39.7% and postulated to be 48% according to Central Veterinary Research Institute (CVRI) and [11]. Consequently, this country has instituted measures for rabies control, encompassing canine immunization, population control, and movement restrictions, as articulated in the Control of Dogs Act within the Laws of Zambia [11].

Despite extant legislation mandating canine vaccination, the districts of Manyinga and Mwansabombwe persistently report suboptimal figures regarding vaccinated canines, unrestricted canine movements, and an escalating incidence of animal and human rabies cases, in addition to canine bites, National Livestock Epidemiology and Information Centre (NALEIC) reports. The aforementioned prompted the selection of these two rural districts and also because no such surveys have been conducted in these rural parts of the country. The dog population in Zambia is estimated to be 934,171, as reported by (NALEIC), but this is not conclusive as the precise canine population in Zambia remains elusive, with a purportedly minute fraction undergoing vaccination [11]. Estimated canine populations in Manyinga and Mwansabombwe hover around 2300 and 1500, respectively, with presumed low vaccination rates and a paucity of rabies awareness in rural populations.

Despite concerted efforts in mass vaccination endeavors, the scope of coverage remains wanting in the country, with the number of notified dog bite cases rising. The Zambian report on rabies was presented at the Southern and East African Rabies Group (SEARG) meeting of 2013; the number of notified dog bite cases in Zambia rose from 620 in 2010 to 732 in 2011 [11]. According to veterinary records, between 2018 and 31 July 2020, only 752 canines in Manyinga and 75 in Mwansabombwe received vaccinations. Primarily concentrated in urban enclaves, these vaccinations neglect the substantial rural canine populace, commonly deployed for illicit hunting and thereby posing substantial health risks to both imperiled wildlife and local denizens. The two districts record dog bite cases every year, but the extent of canine rabies cases in Manyinga and Mwansabombwe remains indeterminate, as ostensibly rabid canines are expeditiously euthanized without postmortem and confirmation by CVRI and the University of Zambia School of Veterinary Medicine laboratory, which is the only laboratory that conducts rabies confirmation in the country. The aforementioned has contributed to the poor rabies surveillance in Zambia, together with the lack of collaboration between human and animal health sectors [12].

The rabies suspected cases analysis recorded in Zambia between 1985 to 2004 found 1088 rabies-positive samples from various species, of which 747 were from dogs and 98 were from humans [10,11]. Another analysis conducted on brain samples collected from suspected rabid dogs between January 2005 and December 2013 found 153 rabies-positive cases [13]. The aforementioned indicates that the dog-mediated human rabies and dog rabies burdens in the country are still a challenge.

This study’s main objective was to identify barriers to rabies prevention and control in the Manyinga and Mwansabombwe districts. Methodologically, the research interrogated the knowledge, attitudes, and practices concerning rabies within the general populace and pet owners. Additionally, it scrutinized demographic facets of the canine populace, estimated the number of vaccinated dogs to gauge mass vaccination coverage, and discerned factors influencing the viability of rabies control programs in the Manyinga and Mwansabombwe districts. By elucidating these impediments, the study aspires to augment canine rabies vaccination coverage to align with the 70% benchmark proposed by the World Health Organization [9], thereby ameliorating attitudes and knowledge among the rural population regarding rabies. 

## 2. Materials and Methods

### 2.1. Study Area and Population

**Figure 1:**
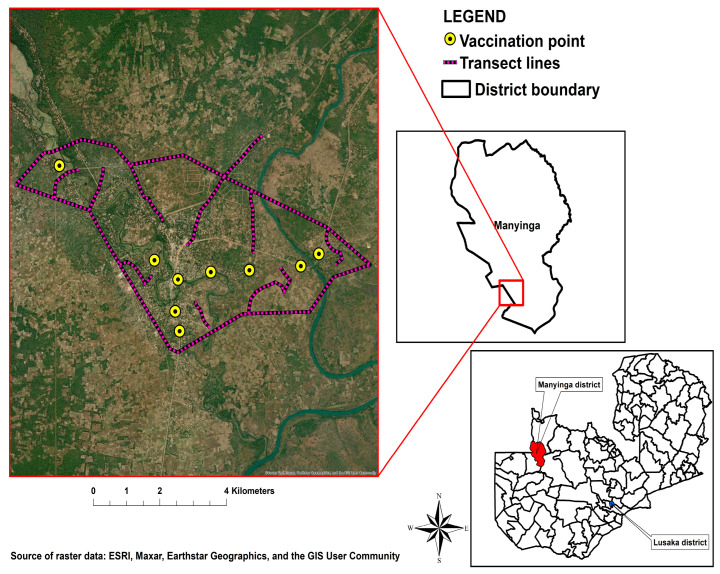
Manyinga district study area.

**Figure 2:**
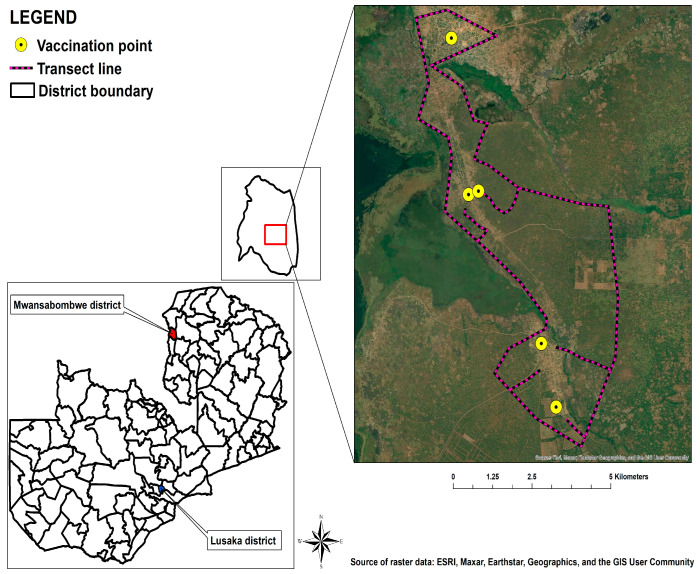
Mwansabombwe district study area.

The study was conducted within the geographic confines of the Manyinga district, situated in the Northwestern Province, and the Mwansabombwe district, located in the Luapula Province of Zambia. Manyinga is positioned at an approximate distance of 331 km from the provincial capital, Solwezi, and 950 km from the national capital, Lusaka (Figure 1). Having attained district status a mere eight years ago, it represents a recent addition to the administrative jurisdiction. Encompassing a landmass of around 21,851 km^2^, the district predominantly features woodlands and streams, constituting 90 percent of its total terrain. Mwansabombwe, situated approximately 939.5 km from the national capital, Lusaka, is an equally nascent administrative unit, having undergone separation from the Kawambwa district in 2012 (Figure 2). This district covers a total expanse of 5252 km^2^. 

The two study districts primarily consist of rural settlements, making it challenging to precisely estimate household numbers. However, based on the 2021 census data, it was approximated that Manyinga has around 14,000 households with a human population of 75,030, whereas Mwansabombwe has 35,546 households. The Manyinga district encompasses four distinct ethnic groups: Luvale, Lunda, Kalunda, and Chokwe. Notably, the Luvale ethnic group is indigenous to Manyinga. In contrast, the primary ethnic group in the Mwansabombwe district is Lunda. Agriculture serves as the predominant livelihood in Manyinga, with both livestock and crop farming playing central roles. Conversely, Mwansabombwe relies heavily on fishing and crop farming as the primary sources of sustenance.

### 2.2. Study Design

A cross-sectional study was used that included household questionnaires, interviewing key informants, and evaluation of rabies vaccination and dog bite case records.

### 2.3. Ethical Aspects

The study was approved by the board of graduate studies (School of Veterinary Medicine, University of Zambia) and ethical clearance was granted by ERES CONVERGE IRB (Ref.No.2021-Jun-023). Participants in the questionnaire survey were told the purpose of the study and consent for participation was obtained. 

### 2.4. Data Collection

#### 2.4.1. Household Surveys

- Pre-sensitization and vaccination household survey

Households were randomly selected using cluster design because of how widely dispersed households are in the Manyinga central camp and Mwansabombwe districts. Then, the households were divided into clusters from the formulated clusters random selection was conducted. The households of the randomly selected clusters participated in the questionnaire survey regardless of whether they owned a dog/cat or not. In all selected households, heads of the household were interviewed; if absent, a suitable substitute of 15 years of age and above was chosen and those without such individuals were omitted from the questionnaire survey. All respondents to the questionnaire were informed of the purpose of the study and consent for participation was obtained.

- Post-sensitization and vaccination household survey

Five months after the mass vaccination campaign, a follow-up questionnaire survey was undertaken to assess the efficacy of the sensitization efforts and rabies awareness. The survey was conducted independently from the first survey participants and it was only conducted in Manyinga because of financial constraints. The goal was to evaluate the impact of the initial mass vaccination on enhancing rabies knowledge among the residents of Manyinga. Throughout the intervening five-month period between the first mass vaccination and the second questionnaire survey, monthly rabies awareness initiatives were implemented. These initiatives were disseminated through the local radio station and community leaders. 

#### 2.4.2. Rabies Sensitization and Awareness 

Rabies sensitization and awareness campaigns were conducted across the districts for 3 months following the initial questionnaire survey. The sensitization campaigns covered what rabies was, its transmission, animals affected, signs of rabies in animals, steps to take when bitten by a dog, rabies prevention/control, dog registration, and responsible dog ownership. These campaigns utilized local radio stations and disseminated posters translated into the local language to promote better acceptance and facilitate a clearer understanding of the presented message. The posters were strategically displayed in various locations, including schools, clinics, markets, churches, residences of village headmen, and all communal gathering points. This comprehensive approach aimed to maximize outreach and engage diverse segments of the community in fostering awareness and understanding of rabies prevention.

#### 2.4.3. Mass Vaccination Campaign

- Transect survey to Estimate Dog and Cat Population

The transect survey was executed by two groups, each comprising three individuals, traversing through the community on foot. The survey areas were demarcated into 2 km by 2 km polygons. To prevent the inclusion of migrating dogs, a 50 m buffer was established around the boundaries within each transect area. The survey involved counting all encountered dogs along the transect line, following the methodology outlined by [12]. Visual identification was employed to estimate the populations of both dogs and cats. Throughout the transect survey, GPS tracking devices were utilized to log movement data. Subsequently, the length of the transect lines was measured using Google Earth Pro software, utilizing the records from the GPS log.

- Mass Vaccination Campaign

A rabies mass vaccination campaign was orchestrated through radio announcements and the dissemination of multilingual posters (English, Luvale, Lunda, and Bemba) in various community settings, including schools, markets, churches, clinics, village headmen houses, and other communal meeting places. The campaign featured multiple central vaccination points, notably in Manyinga at the central veterinary camp and in Mwansabombwe at the veterinary camps.

During the 21-day mass vaccination campaign in both districts, dogs and cats underwent vaccination with 1 mL of Rabies Vet (Bio-Med Private Ltd., Ghaziabad, India) intramuscularly. Each dog or cat was administered the vaccine using a new needle and syringe to ensure hygiene and prevent contamination. Following vaccination, all dogs and cats were distinctly marked with red-colored spray on their bodies to prevent re-vaccination and save as an identification feature for vaccinated dogs and cats. Additional details such as sex, age, color markings, owner information, and previous vaccination records were collected, followed by the issuance of a new vaccination certificate. This method facilitated the easy identification of vaccinated dogs and cats, distinguishing them from those that remained unvaccinated.

- Transect survey (re-capture)

Two days following the mass vaccination, a transect survey (re-capture) was undertaken in the Manyinga and Mwansabombwe districts to assess the uptake of rabies vaccines. Two groups conducted the transect survey, utilizing motorbikes. During the survey, the teams systematically counted all encountered dogs along the transect lines, distinguishing between those that had been sprayed (marked) and those that had not, in accordance with the methodology outlined by Kaneko et al. [12]. Through visual identification of vaccinated dogs, an estimate of the coverage of rabies mass vaccinations was then determined.

#### 2.4.4. Interviewing Key Informants

The second phase of the survey involved interviewing key informants. This comprised conducting in-depth interviews with local rabies experts, including representatives from the District Veterinary Department and Rabies Control Officers from the district council and Ministry of Health public health officers. The key informants were asked if they knew what rabies was, its transmission, and prevention and control. Furthermore, the role the officers played in the prevention and control of rabies was probed, as well as the challenges they faced in implementing the prevention and control measures. 

#### 2.4.5. Assessment of Dog and Jackal Bite Cases 

An evaluation of rabies vaccination and dog bite case records was conducted at both the veterinary offices and district hospitals. This assessment aimed to determine the frequency of dog bites and the vaccination status of dogs involved in reported bite incidents.

### 2.5. Data Analysis

The collected data were entered into a Microsoft Excel spreadsheet and subsequently exported to IBM SPSS Statistics version 26.0 for a comprehensive thematic analysis. Descriptive statistics were generated, and cross tabulations calculating Pearson’s Chi Square were performed in comparing demographic variables of Manyinga pre- and post-sensitization and testing of association on demographic knowledge and rabies knowledge.

## 3. Results

### 3.1. Demographic Characteristics of Respondents from Mwansabombwe and Manyinga

A total of 301 and 100 representing a response rate of 78.0% and 26.2% in Manyinga and Mwansabombwe were interviewed, respectively. The poor response was observed in Mwansabombwe due to refusals. From the surveys conducted in the two districts, there was a higher participation of females. In both districts, the majority of respondents had attained some form of formal education. Specifically, in Mwansabombwe, most respondents were farmers, while in Manyinga, respondents were predominantly students. Moreover, Mwansabombwe had fewer respondents from households owning pets compared to Manyinga (Table 1).

### 3.2. Rabies Awareness and Knowledge in Manyinga and Mwansabombwe

In terms of rabies knowledge, 88.0% of respondents from both districts had prior awareness of rabies. Notably, Mwansabombwe respondents exhibited a higher level of information regarding prevention (82.0%), signs of rabies in animals (77.0%), and signs of rabies in humans (69.0%). Concerning the mode of transmission, Mwansabombwe respondents displayed greater knowledge, with 97.7% identifying bites as the primary mode, compared to 95.6% in Manyinga.

Regarding the source of rabies information, respondents from both districts predominantly cited veterinarians. Mwansabombwe’s veterinarians and health workers were particularly effective in rabies sensitization, being more exposed to television and radio compared to Manyinga. However, they were less exposed to social media and other information sources (Table 2).

### 3.3. Demographics, Knowledge, and Attitude of Dog Owners toward Rabies Prevention and Control Manying and Mwansabombwe

Among respondents who owned dogs, the majority fell within the age range of 15–25 years in Manyinga; in Mwansabombwe, they were predominantly between 36–45 years old, female, and residing in rural areas (65.9% and 48.8% in Manyinga and Mwansabombwe, respectively). Additionally, a significant proportion in both districts had at least attained a primary level of education. Regarding occupation, the majority were students in Manyinga, whereas farmers were predominant in Mwansabombwe.

With regards to respondents who owned dogs, the majority demonstrated awareness of rabies, knowledge about signs of rabies in animals, prevention and control measures, the mode of transmission, signs of rabies in humans, and adherence to pet vaccination. Mwansabombwe had a higher number of well-informed participants compared to Manyinga. Overall, dog owners in Mwansabombwe exhibited a robust knowledge of rabies, and their attitude towards prevention and control was commendable (Table A1).

### 3.4. Differences in Demographic Characteristics and Rabies Knowledge among Respondents in Mwansabombwe and Manyinga

Regarding age, respondents aged 31–45 years in Mwansabombwe and respondents aged 26–35 years in Manyinga demonstrated higher awareness of rabies. Additionally, respondents aged 46 years and above were more likely to be aware of rabies in both districts. In terms of gender, males exhibited greater awareness of rabies in Mwansabombwe, while in Manyinga, both males and females were equally aware. According to settlement, respondents from semi-urban and urban areas displayed higher awareness compared to those from rural areas in both districts. Concerning education, respondents who had attained tertiary education showed greater awareness of rabies in both Mwansabombwe and Manyinga.

In Mwansabombwe, a significant relationship between settlement (*p* = 0.007) and age (*p* = 0.001) with rabies knowledge was observed in terms of rabies awareness. However, gender (*p* = 0.203), occupation (*p* = 1.97), and education (*p* = 0.071) exhibited no significant relationship with rabies knowledge. On the other hand, in Manyinga, there was a significant relationship between education (*p* = 0.011) and occupation (*p* = 0.044) with rabies knowledge, while gender (*p* = 0.995), age (*p* = 0.516), and settlement (*p* = 0.133) had no significant relationship with rabies knowledge.

### 3.5. Demographic Characteristics of Respondents in Manyinga Pre-Sensitization vs. Post-Sensitization

A total of 301 individuals participated in both the pre-sensitization and post-vaccination surveys in the Manyinga district. In both surveys, the majority of respondents were female, had acquired some level of formal education, resided in rural areas, and were pet owners (Table 3).

A comparison of the demographic variables between the two groups pre-sensitization and post-sensitization revealed that there was no significance in terms of age (*p* = 0.970), gender (*p* = 0.633), settlement (*p* = 0.503), education (*p* = 0.961), and occupation (*p* = 0.514) in both surveys, as shown in Table 3.

### 3.6. Rabies Awareness and Knowledge

In relation to pre-sensitization knowledge, the majority (88.0%) of respondents in Manyinga were already familiar with rabies. They demonstrated awareness of prevention methods, the mode of transmission, could identify at least one animal affected by rabies, and were acquainted with signs of rabies in humans. However, there was a gap in recognizing signs of rabies in animals. The primary source of rabies information for the majority of respondents was veterinarians.

Post-sensitization, there was a notable improvement in knowledge. The majority (90.4%) of respondents were now informed about rabies, including prevention methods, the mode of transmission, identification of animals affected by rabies, and recognition of signs of rabies in animals, although there was a decrease in the awareness of signs in humans. Veterinarians remained a predominant source of information, cited by 82.2% of respondents (Table 4).

Overall, a positive shift in respondents’ knowledge was observed after sensitization and mass vaccinations, particularly in terms of having heard about rabies, and preventing and recognizing signs of rabies in animals (Table 4).

### 3.7. Dog Demographics, Knowledge, and Attitude of Dog Owners toward Rabies Prevention and Control Pre- and Post-Sensitization in Manyinga

Among respondents who owned dogs, the majority were between the age range of 15–25 years, female, rural based, 65.9% and 60.7% pre- and post-sensitization, respectively, and had at least attained a secondary level of education in both surveys. With regards to occupation during the pre-sensitization survey, the majority were students, while the majority were farmers in post-sensitization.

In the pre-sensitization survey, the majority (89.1%) were already aware of rabies, possessed knowledge about prevention and control measures, identified modes of transmission, and were familiar with the signs of rabies in humans. Moreover, they were informed about the importance of pet vaccination (Table A2).

Post-sensitization, the knowledge and attitude of dog owners toward rabies prevention and control remained consistent. The majority (94.2%) continued to demonstrate awareness of rabies, knowledge about prevention and control, identification of modes of transmission, recognition of clinical signs in animals, and adherence to pet vaccination practices. The overall knowledge and attitude of dog owners regarding rabies and its prevention and control remained largely unchanged in both surveys (Table A2).

### 3.8. Differences in Demographic Characteristics and Rabies Knowledge among Respondents Pre- and Post-Sensitization in Manyinga

In both surveys, it was observed that respondents aged between 26 and 35 years exhibited greater awareness of rabies compared to those between 15 and 25 years of age. Additionally, respondents aged 46 and above were more likely to be aware of rabies. Among the gender demographic, the majority of males demonstrated a higher level of awareness of rabies. Urban-based respondents were found to be more aware, and individuals with tertiary education showed a higher level of awareness regarding rabies (Table 5).

In the pre-vaccination survey, a noteworthy association between education (*p* = 0.011) and occupation (*p* = 0.044) with rabies knowledge was identified. However, gender (*p* = 0.995), age (*p* = 0.516), and settlement (*p* = 0.133) exhibited no significant relationship with rabies knowledge.

In the post-sensitization rabies awareness survey, a significant relationship was observed between education (*p* = 0.005) and occupation (*p* = 0.006) with rabies knowledge. Conversely, no significant relationship was found with gender (*p* = 0.062), age (*p* = 0.148), and settlement (*p* = 0.999). Throughout both surveys in the Manyinga district, education and occupation consistently demonstrated a significant relationship with rabies knowledge (Table 5).

### 3.9. Key Informants View on Rabies

The interviews with key informants involved 10 participants, of which 2 were females. Veterinary staff were asked what they know about rabies, its prevention and control, the perspective of their communities about rabies, and the challenges being faced in preventing and controlling it. The council public health officers were asked about the challenges they are facing in enforcing the law, as well as public health officers’ challenges facing the prevention of human rabies. Informants from both districts were well vested with rabies knowledge and they revealed that a significant portion of the population was aware of rabies and the primary preventive measures in place, but there was a prevalent desire for vaccinations to be provided free of charge. The departments highlighted that the control of rabies faced obstacles such as inadequate staff and insufficient resources. Additionally, challenges in dog handling and control resulted in a limited number of dogs being presented during vaccination campaigns.

Implementing the dog control law by the local authority faced difficulties, particularly with dog registration and dog movement control. Few houses were enclosed in fences, and a limited number of dog owners were able to restrain their dogs, leading to dogs scavenging at dump sites that are near residential areas. This situation made it challenging to differentiate owned dogs from stray dogs. The reviews indicated that responsible dog ownership and movement restrictions were not effectively implemented, as owned dogs could not be easily distinguished from unowned dogs.

The public health department alluded to the unavailability of post-exposure prophylaxis (PEP) in hospitals being a major challenge and inadequate staff to conduct sensitizations in the communities on action to be taken in case of a dog bite. The non-availability of PEP in government hospitals made it expensive for dog bite victims to get PEP because they had to procure it on their own or travel to neighboring districts, which necessitated incurring transport and procurement costs, making it expensive for poor victims.

The follow-up on suspected rabies cases proved challenging due to community practices involving the killing and disposal of suspected rabid dogs in rivers after biting incidents. Furthermore, the absence of laboratories in districts and provincial head offices capable of testing for rabies added to the complexities of managing and confirming rabies cases.

### 3.10. Evaluation of 2017 to 2022 Dog Bite Cases

The assessment of dog bite cases from the veterinary office and Loloma Mission Hospital revealed a total of 538 recorded cases spanning from January 2017 to June 2022. The ages of the victims varied between 3 and 56 years. Notably, in Manyinga, 75.0% of the recorded cases involved victims below the age of 15 years. Unfortunately, in Mwansabombwe, the age range of victims could not be determined due to a lack of available records.

**Figure 3:**
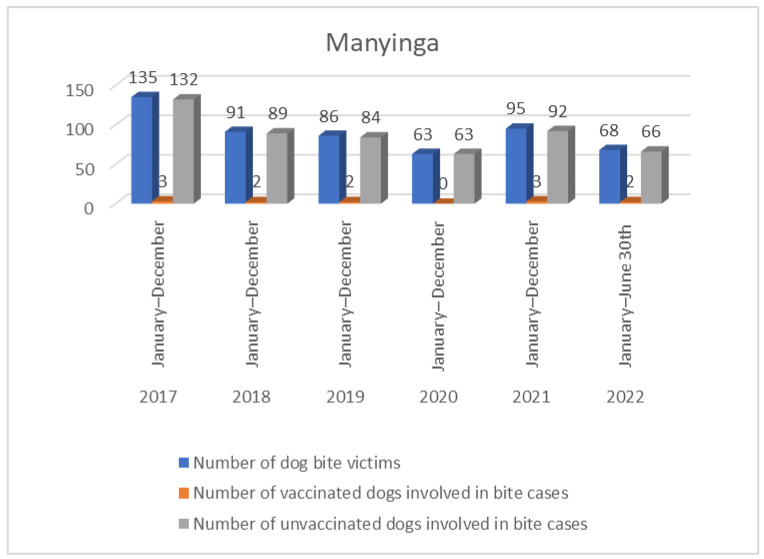
Manyinga dog bite records from January 2017–30th June 2022.

**Figure 4:**
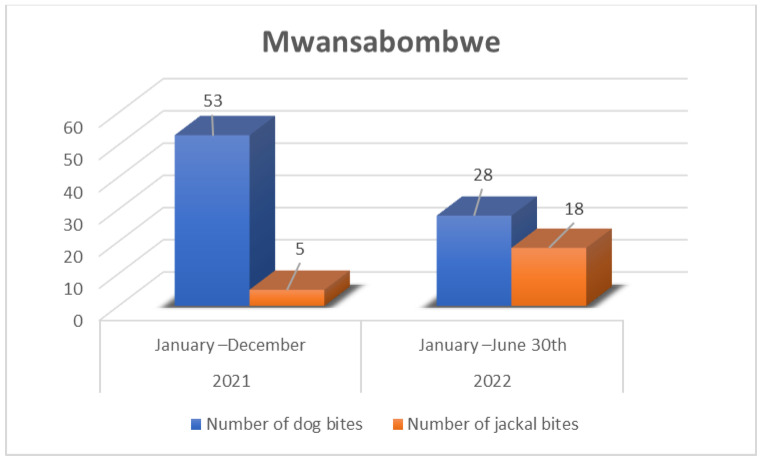
Mwansabombwe dog and jackal bites record from January 2021–30th June 2022.

In Manyinga district, 55.0% of the bite cases, and in Mwansabombwe, all cases, were attributed to dogs whose owners were unknown, making it impossible to ascertain the vaccination status of these dogs. Of these cases, 43.0% were linked to owned but unvaccinated dogs, while a mere 2.0% resulted from bites by vaccinated dogs with valid vaccination certificates (Figure 3 and Figure 4). This aligns with the veterinary records in the Manyinga district, where the validity of vaccination certificates was determined based on the occurrence of the bite preceding the next scheduled dog vaccination.

Victims of bites from unvaccinated dogs were advised by the district veterinary officer to undergo wound management, tetanus treatment, and post-exposure prophylaxis. All recorded victims of bites from unvaccinated dogs, except for two individuals, received the recommended treatment and post-exposure vaccines. Regrettably, the two individuals who did not receive post-exposure vaccines succumbed to the disease in 2018 at Loloma Mission Hospital.

### 3.11. Dog Vaccination Coverage

A total of 481 dogs received vaccinations at nine designated points within the Manyinga central veterinary camp from 27 May 2022 to 2 June 2022. In Mwansabombwe, 326 dogs were vaccinated at five designated points across the three veterinary camps. The vaccination coverage in the study area reached 481 out of an estimated 750 dogs, representing 64% of the dog population in Manyinga. In Mwansabombwe, the coverage was 326 out of an estimated 1500 dogs, representing 21.0% of the dog population. Notably, in both study areas, the targeted 70.0% vaccination rate, as recommended by WHO, was not achieved.

## 4. Discussion

This study represents a pioneering effort in both the Manyinga and Mwansabombwe districts of Zambia concerning rabies control. This study is the first of its kind in both the Manyinga and Mwansabombwe districts, and aims to comprehensively assess the knowledge, attitudes, and practices related to rabies, with a specific focus on the prevention and control of this zoonotic disease. The primary objectives encompassed exploring the general understanding of rabies among the residents, evaluating the demographics and vaccination status of the dog population, and identifying barriers to the successful implementation of rabies control programs in these districts.

The collected data reveals that 88.0% of respondents from both districts had heard about rabies. Before sensitization in Manyinga, only 29.9% knew the signs of rabies in animals, but after sensitization and vaccination, this percentage increased to 54.5%. In Mwansabombwe, 77.0% of respondents knew about rabies signs. However, the majority remained unaware of the signs of rabies in dogs and humans. Comparing our study with others, a study in Lilongwe, Malawi, reported that 98.0% of respondents had heard of rabies, with 71.0% aware of its transmission from dogs to humans [14]. The improvement in rabies knowledge among respondents was a result of sensitization campaigns that covered what rabies was, its transmission, animals affected, signs of rabies in animals, steps to take when bitten by a dog, rabies prevention/control, dog registration and responsible dog ownership. This study recorded a higher proportion of female respondents, possibly due to cultural practices where females remain at home while males go to work, farm, and dominate educational meetings, hence their absence from home to take part in the survey. The study also showed that the rural community had more dogs than the urban community, which is similar to the study conducted in Nyimba Zambia, Kwazulu-Natal, South Africa, and Mutendere, Zambia [11,15,16]. Although dog owners demonstrated good knowledge of rabies in both districts, additional sensitization efforts from the veterinary department, medical health, and council public health workers are crucial for imparting accurate knowledge. This enhanced awareness can lead to responsible dog ownership and emphasize the importance of rabies vaccination. Official education by experts may improve dog handling skills and subsequently increase vaccination coverage to effectively control rabies outbreaks [12].

Research findings underscore the common occurrence of dog bite/jackal bite cases in both study areas. Although unreported cases were not determined, studies from Nyimba and Tanzania suggest that for every reported case, at least 10 cases go unreported [11,17]. Notably, 55.0% of dog bite cases in this study involved unknown dogs, highlighting the challenge of absent dog registration and restricted movements, making proper identification difficult. This situation complicates the differentiation between owned and stray dogs. In contrast, a study in South Africa’s KwaZulu-Natal found that 83.0% of dog bite cases were caused by owned dogs [18].

In our study, 70.0% of dog bites were attributed to unknown dogs, and 75.0% of victims were below 15 years old, aligning with global trends identified by the World Health Organization. The proximity of garbage dumping sites to residential areas may contribute to this phenomenon because of the lack of barriers around these sites. This age group tends to play at these dump sites and is seen deliberately provoking the scavenging dogs as a game, which leads to dog bites.

For victims of dog bites of dogs of unknown ownership which were considered to be unvaccinated dogs, post-exposure prophylaxis (PEP) was recommended. However, PEP availability varied between Manyinga and Mwansabombwe, impacting accessibility and incurring additional costs for transportation. Vaccinating dogs could reduce such costs, as studies indicate that dog vaccination is more cost-effective than PEP for rabies control [11]. Lack of law enforcement by the local government and irresponsible dog ownership are prevalent in Manyinga and Mwansabombwe, hindering the differentiation between owned and stray dogs. This complicates rabies control efforts, requiring community compliance through responsible dog ownership. The lack of fencing in the majority of households allows for uncontrolled dog movement and breeding, resulting in more stray dogs. Challenges include the absence of dog registration due to attached fees and various obstacles hindering a coordinated global approach to canine rabies elimination [19].

Vaccination coverage in both study areas fell short of the WHO-recommended 70%, with 64% in Manyinga and 21% in Mwansabombwe. This is lower than reported coverage in rural Mazabuka [12], and rural areas of Tanzania Mara and Serengeti [20,21]. The findings suggest that ordinary vaccination coverage in rural Zambia does not reach the critical threshold of 20.0–45.0% needed to interrupt dog rabies transmission [12], but in this study the critical threshold in the study areas was reached after the vaccination campaign indicating that it is attainable.

Dog owners identified financial constraints, distance to veterinary offices, vaccine unavailability, and certain beliefs as reasons for not vaccinating their dogs. Overcoming these barriers requires addressing inadequate veterinary staffing, securing funding for vaccine procurement, and dispelling misconceptions within the community. A study by Sivagurunathan et al. [22] and Nejash et al. [23] emphasizes that rabies cases predominantly occur in poor communities characterized by poor dog ownership and an unwillingness or inability to pay for vaccinations.

Our study faced limitations, including insufficient resources for conducting a second post-sensitization questionnaire survey in Mwansabombwe. This hindered our ability to assess the impact of sensitization and vaccination on rabies knowledge. Additionally, there was reluctance among Mwansabombwe residents to participate in the survey. Achieving the WHO-recommended 70.0% vaccination coverage was hindered by various challenges. These challenges encompassed owners’ reluctance to handle their dogs, prevailing community beliefs such as the misconception that vaccinating a dog hinders its hunting abilities, and insufficient resources to sustain the vaccination campaign over an extended period.

## 5. Conclusions

The study reveals a formidable challenge in controlling rabies in Manyinga and Mwansabombwe due to existing barriers. However, potential solutions include knowledge dissemination and free mass vaccination campaigns for owned dogs. Despite efforts, both districts fell short of the WHO’s 70% coverage recommendation. To overcome these challenges, essential interventions are required. Community education on rabies can foster responsible dog ownership, reducing stray dogs and improving vaccination coverage. This aligns with the WHO’s goal of zero rabies cases by 2030. Addressing staffing issues at veterinary offices and ensuring rabies vaccinations are accessible are crucial steps. Identifying these barriers lays the groundwork for targeted interventions toward the global objective of rabies elimination.

## Figures and Tables

**Table 1:** Demographic data of respondents from Mwansabombwe and Manyinga (pre-sensitization).

	MANYINGA		MWANSABOMBWE	
Variables	*N* = 301	%	*N* = 100	%
AGE				
15–25 Years	194	64.5%	20	20.0%
26–35 Years	72	23.9%	23	23.0%
36–45 Years	13	4.3%	27	27.0%
46–55 Years	12	4.0%	19	19.0%
56 Years and above	10	3.3%	11	11.0%
GENDER				
Male	142	47.2%	42	42.0%
Female	159	52.8%	58	58.0%
EDUCATION LEVEL				
Primary	4	1.3%	34	34.0%
Secondary	254	84.4%	28	28.0%
Tertiary	37	12.3%	27	27.0%
Others	6	2.0%	11	11.0%
SETTLEMENT				
Urban	22	7.3%	4	4.0%
Semi-urban	79	26.2%	42	42.0%
Rural	200	66.4%	54	54.0%
OWN PET				
Yes	220	73.1%	43	43.0%
No	81	26.9%	57	57.0%
OCCUPATION				
Government employee	15	5.0%	16	16.0%
Student	227	75.4%	15	15.0%
Farmer	31	10.3%	43	43.0%
Others	28	9.3%	26	26.0%

**Table 2:** Rabies awareness and knowledge in Manyinga and Mwansabombwe.

Questions	MANYINGA		MWANSABOMBWE	
	*N*	%	*N*	%
Have you heard of Rabies?				
YES	265	88.0%	88	88.0%
NO	36	12.0%	12	12.0%
Do You know signs of Rabies in Animals?				
YES	90	29.9%	77	77.0%
NO	211	70.1%	23	23.0%
Do you know how rabies is prevented/controlled?				
YES	183	60.8%	82	82.0%
NO	118	39.2%	18	18.0%
Do you know signs of rabies in humans?				
YES	162	53.8%	69	69.0%
NO	139	46.2%	31	31.0%
Do you know animals that get infected with rabies?				
COW	65	24.0%	14	15.9%
DOG	239	90.2%	86	97.7%
CAT	108	40.8%	58	65.9%
GOAT	47	17.7%	11	12.5%
SHEEP	30	11.3%	11	12.5%
PIG	71	26.8%	11	12.5%
FOX	73	27.5%	51	58.0%
What was your source of Rabies information?				
Veterinary	82	31.3%	51	58.0%
Health workers	67	25.6%	34	38.6%
Television	47	17.9%	28	31.8%
Radio	18	6.90%	25	28.4%
Social media	37	14.1%	5	5.7%
Others	63	24.0%	7	8.0%
How is Rabies transmitted?				
Bites	208	83.2%	86	97.7%
Scratch	15	6.0%	13	14.8%
Contact saliva	17	6.8%	31	35.2%
Cuts	14	5.6%	19	21.6%
Uncooked Meat	6	2.4%	4	4.5%
Urine	2	0.8%	2	2.3%

**Table 3:** Demographic characteristics of respondents pre- and post-sensitization in Manyinga.

Variable		Pre-Sensitization (%)	Post-Sensitization (%)	*p*-Value
Age	15–25 years 26–35 years 36–45 years 46–55 years 56 years and above	194 (64.5%) 72 (23.9%) 13 (4.3%) 12 (4.0%) 10 (3.3%)	191 (63.5%) 56 (18.6%) 20 (6.6%) 27 (9.0%) 7 (2.3%)	0.970
Gender	Male Female	142 (47.2%) 159 (52.8%)	144 (47.8%) 157 (52.2%)	0.633
Settlement	Urban Semi-urban Rural	63 (20.9%) 79 (26.2%) 159 (52.8%)	24 (8.0%) 84 (27.9%) 193 (64.1%)	0.503
Education	Primary Secondary Tertiary Others	41 (13.6%) 182 (60.5%) 48 (15.9%) 30 (10.0%)	53 (17.6%) 197 (65.4%) 43 (14.3%) 8 (2.7%)	0.961
Occupation	Government Student Farmer Others	50 (16.6%) 165 (54.8%) 39 (13.0%) 47 (15.6%)	19 (6.3%) 78 (25.9%) 132 (43.9%) 72 (23.9%)	0.514

**Table 4:** Rabies awareness of study population pre- and post-sensitization in Manyinga.

Questions	Pre-Sensitization		Post-Sensitization	
	*N*	%	*N*	%
Have you heard of rabies?				
YES	265	88.0%	272	90.4%
NO	36	12.0%	29	9.6%
Do you know signs of rabies in animals?				
YES	90	29.9%	165	54.5%
NO	211	70.1%	138	45.5%
Do you know how rabies is prevented/controlled?				
YES	183	60.8%	207	68.5%
NO	118	39.2%	95	31.5%
Do you know signs of rabies in humans?				
YES	162	53.8%	117	38.7%
NO	139	46.2%	185	61.3%
Do you know animals that get infected with rabies?				
COW	65	24.0%	85	34.4%
DOG	239	90.2%	245	100.0%
CAT	108	40.8%	150	60.7%
GOAT	47	17.7%	75	30.4%
SHEEP	30	11.3%	75	30.4%
PIG	71	26.8%	80	32.4%
FOX	73	27.5%	160	64.8%
What was your source of rabies information?				
Veterinary	82	31.3%	222	82.2%
Health workers	67	25.6%	9	3.3%
Television	47	17.9%	13	4.8%
Radio	18	6.9%	116	43.0%
Social media	37	14.1%	29	10.7%
Others	63	24.0%	9	3.3%
How is rabies transmitted?				
Bites	208	83.2%	236	87.7%
Scratch	15	6.0%	0	0%
Contact saliva	17	6.8%	33	12.3%
Cuts	14	5.6%	0	0%
Uncooked Meat	6	2.4%	0	0%
Urine	2	0.8%	0	0%

**Table 5:** Differences in demographic characteristics and rabies knowledge among respondents pre- and post-sensitization in Manyinga.

HEARD OF RABIES						
VARIABLES	PRE-SENSITIZATION			POST-SENSITIZATION		
	Count	Percent (%)	*p*-Value	Count	Percent (%)	*p*-Value
AGE						
15–25 Years	166	85.0%		141	73.0%	
26–35 Years	67	93.0%	0.516	49	87.5%	0.148
36–45 Years	12	92.0%		16	80.0%	
46–55 Years	11	91.6%		24	88.9%	
56 Years and Above	9	90.0%		7	100.0%	
GENDER						
Male	125	88.0%	0.995	121	83.4%	0.062
Female	140	88.0%		181	74.7%	
EDUCATION LEVEL						
Primary	2	50.0%	0.011	50	94.3%	
Secondary	223	87.8%		146	73.7%	0.006
Tertiary	36	97.0%		35	79.5%	
Others	4	66.6%		8	100.0%	
OCCUPATION						
Government employee	15	100.0%	0.044	19	100.0%	
Student	193	85.0%		53	67.0%	0.006
Farmer	30	96.7%		109	81.9%	
Others	27	96.4%		58	80.5%	
SETTLEMENT						
Urban	22	100.0%	0.133	19	79.1%	
Semi-urban	71	89.8%		67	78.8%	0.999
Rural	172	86.0%		153	78.8%	

## Data Availability

Data presented is available on request from corresponding authors.

## Appendix A

**Table A1:** Demographics, knowledge, and attitudes of dog owners towards rabies prevention and control in Manyinga vs. Mwansabombwe.

VARIABLES	MANYINGA PRE-SENSITIZATION		MWANSABOMBWE	
	Frequency	Percent %	Frequency	Percent %
OWN DOGS				
AGE				
15–25 YRS	149	67.7%	7	16.3%
26–35 YRS	45	20.5%	10	23.3%
36–45 YRS	7	3.2%	13	30.2%
46–55 YRS	12	5.5%	9	20.9%
56 YRS AND ABOVE	7	3.2%	4	9.3%
GENDER				
MALE	103	46.8%	19	44.2%
FEMALE	117	53.2%	24	55.8%
SETTLEMENT				
URBAN	19	8.6%	1	2.3%
SEMI-URBAN	56	25.5%	21	48.8%
RURAL	145	65.9%	21	48.8%
EDUCATION LEVEL				
PRIMARY	1	0.5%	16	37.2%
SECONDARY	188	85.5%	12	27.9%
TERTIARY	25	11.4%	10	23.3%
OTHERS	6	2.7%	5	11.6%
OCCUPATION				
GOVERNMENT EMPLOYEE	8	3.6%	5	11.6%
STUDENT	176	80.0%	6	14.0%
FARMER	21	9.5%	19	44.2%
OTHERS	15	6.8%	13	30.2%
Have you heard of Rabies?				
YES	196	89.1%	40	93.0%
NO	24	10.9%	3	7.0%
Do you know signs of Rabies in Animals?				
YES	75	34.1%	35	81.4%
NO	145	65.9%	8	18.6%
Do you know signs of Rabies in Humans?				
YES	135	61.4%	34	79.1%
NO	85	38.6%	9	20.1%
Is your pet vaccinated?				
YES	131	59.5%	35	81.4%
NO	88	40.0%	8	18.6%
How is Rabies transmitted?				
BITE	178	96.7%	39	97.5%
CUTS	5	2.7%	9	22.5%
SCRATCH	7	3.8%	4	10.0%
CONTACT WITH SALIVA	9	4.9%	16	40.0%
UNCOOKED MEAT	2	1.1%	2	5.0%
URINE	0	0.0%	2	2.5%
Do you know how Rabies is prevented and controlled?				
YES	146	66.4%	41	95.3%
NO	74	33.6%	2	4.7%

## Appendix B

**Table A2:** Demographics, knowledge, and attitudes of dog owners towards rabies prevention and control pre- and post-sensitization in Manyinga.

VARIABLES	OWN DOGS			
	PRE-SENSITIZATION		POST-SENSITIZATION	
	Frequency	Percent %	Frequency	Percent %
AGE				
15–25 YRS	149	67.7%	110	63.6%
26–35 YRS	45	20.5%	30	17.3%
36–45 YRS	7	3.2%	11	6.4%
46–55 YRS	12	5.5%	17	9.8%
56 YRS AND ABOVE	7	3.2%	5	2.9%
GENDER				
MALE	103	46.8%	84	48.6%
FEMALE	117	53.2%	89	51.4%
SETTLEMENT				
URBAN	19	8.6%	14	8.1%
SEMI-URBAN	56	25.5%	54	31.2%
RURAL	145	65.9%	105	60.7%
EDUCATION LEVEL				
PRIMARY	1	0.5%	36	20.8%
SECONDARY	188	85.5%	104	60.1%
TERTIARY	25	11.4%	26	15.0%
OTHERS	6	2.7%	7	4.0%
OCCUPATION				
GOVERNMENT EMPLOYEE	8	3.6%	13	7.5%
STUDENT	176	80.0%	43	24.9%
FARMER	21	9.5%	78	45.1%
OTHERS	15	6.8%	39	22.5%
Have you heard of Rabies?				
YES	196	89.1%	163	94.2%
NO	24	10.9%	10	5.8%
Do you know signs of Rabies in Animals?				
YES	75	34.1%	113	65.3%
NO	145	65.9%	60	34.7%
Do you know signs of Rabies in Humans?				
YES	135	61.4%	98	56.6%
NO	85	38.6%	75	43.4%
Is your pet vaccinated?				
YES	131	59.5%	152	87.9%
NO	88	40.0%	21	12.1%
How is Rabies transmitted?				
TRNS BITE	178	96.7%	152	100%
TRNS CUTS	5	2.7%	0	0%
TRNS SCRATCH	7	3.8%	0	0%
TRNS CONTCT SALIVA	9	4.9%	23	15.1%
TRNS UNCOOKED MEAT	2	1.1%	0	0%
TRNS URINE	0	0.0%	0	0%
Do you know how Rabies is prevented and controlled?				
YES	146	66.4%	155	89.6%
NO	74	33.6%	18	10.4%
